# The Therapeutic Potential of MicroRNAs in Atrial Fibrillation

**DOI:** 10.1155/2020/3053520

**Published:** 2020-03-12

**Authors:** Xiaona Xu, Zhiqiang Zhao, Guangping Li

**Affiliations:** Tianjin Key Laboratory of Ionic-Molecular Function of Cardiovascular Disease, Department of Cardiology, Tianjin Institute of Cardiology, The Second Hospital of Tianjin Medical University, Tianjin 300211, China

## Abstract

One of the most globally prevalent supraventricular arrhythmias is atrial fibrillation (AF). Knowledge of the structures and functions of messenger RNA (mRNA) has recently increased. It is no longer viewed as solely an intermediate molecule between DNA and proteins but has come to be seen as a dynamic and modifiable gene regulator. This new perspective on mRNA has led to rising interest in it and its presence in research into new therapeutic schemes. This paper, therefore, focuses on microRNAs (miRNAs), which are small noncoding RNAs that regulate posttranscriptional gene expression and play a vital role in the physiology and normative development of cardiovascular systems. This means they play an equally vital role in the development and progression of cardiovascular diseases. In recent years, multiple studies have pinpointed particular miRNA expression profiles as being associated with varying histological features of AF. These studies have been carried out in both animal models and AF patients. The emergence of miRNAs as biomarkers and their therapeutic potential in AF patients will be discussed in the body of this paper.

## 1. Introduction

It is well known that RNA can be edited and modified, that RNA can form both secondary and tertiary structures, and that RNA experiences a dynamic, tight, and occasionally reversible posttranscriptional regulation through various RNA-binding proteins. Because of this knowledge, biotechnology companies are currently undertaking the clinical development of RNA-targeting therapies. It is in the companies' interests to increase the number of “drug-able” targets. One such target biotechnological research has been the endogenic regulators of gene silencing, microRNAs (miRNAs). They have been investigated due to their potential as therapeutic agents [1]. Initially found in *Caenorhabditis elegans* (*C. elegans*) in 1993, miRNAs are known for using messenger RNA (mRNA) degradation and translational repression to inhibit their target genes [2]. miRNAs regulate gene expression on a posttranscriptional level. They are short, noncoding RNAs, which can bind mRNAs and regulate gene expression through either mRNA degradation or translational repression [3]. mRNA degradation and the blockage of mRNA translation are the two potential mechanisms for miRNA repression of gene expression [4]. Additionally, more than one miRNA has arrhythmogenic potential, and there are always different miRNAs acting within different types of atrial fibrillation (AF) [5]. Thus, to be stable, specific, and potent and have low levels of toxicity, RNA-targeting therapeutic modalities require different chemical modifications.

AF is the most common cardiac arrhythmia, correlated with increased morbidity and mortality rates [6]. Although multiple novel molecular concepts of AF pathophysiology have been in development throughout the previous decade, most of the therapeutic approaches presently available have major limitations. These include a lack of potency and negative side effects, such as malignant arrhythmias in the ventricle [7]. The genetic programming of miRNA regulations, both downregulation and upregulation, has been shown to affect developmental changes [8]. However, AF and other multiple cardiovascular diseases, which lead to myocardial remodeling, have been associated with changes (due to altered miRNA expression levels) in circulating blood and in cardiovascular tissues [9, 10]. miRNAs have been identified as active elements in multiple cardiovascular diseases. This is what motivates further research into their role in AF pathophysiology, as the study of miRNAs could lead to the successful development of safer treatment options with higher efficacy [11, 12].

This review is aimed at providing a summary of the most recent developments in miRNA research based on both human and animal models. Firstly, the studies currently available, which have investigated the role of miRNAs in AF, will be described. This will provide a basis for suggestions as to the extent to which miRNAs might have the capacity to positively affect therapeutic strategies. Once an expression profile of miRNA on the development of AF is given, taking into consideration both human and animal studies, the potential roles of miRNA in AF regulation will be discussed. Following this, the potential function of miRNAs in AF's pathophysiological processes will be considered in accordance with the available experimental evidence. In this way, the possible future clinical applications of miRNAs in AF will be expressed.

## 2. Pathomechanisms of AF

**Figure 1:**
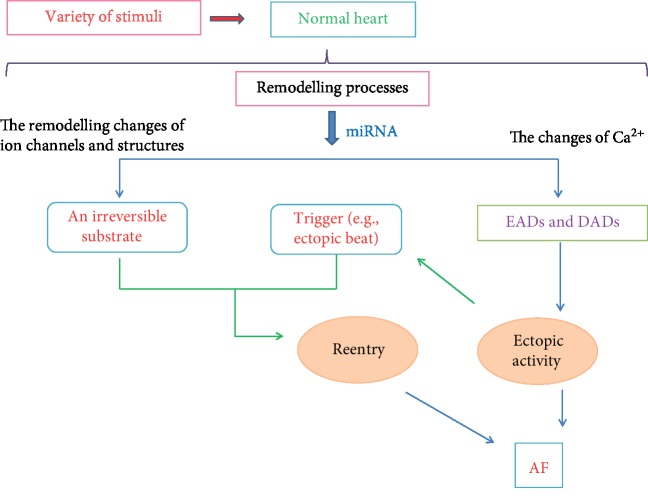
Illustration of AF mechanisms. Reentrant activity and ectopic activity are contained within the pathogenesis. Both the structure and function of the atrial tissue will be altered after the normal heart is stimulated. A substrate for reentrant AF is created by atrial remodeling through the alteration of ion-channel function or the introduction of tissue fibrosis. The likelihood of ectopic activity can be increased by remodeling through the production of Ca^2+^ in cell handling, which can cause triggered activity. Abbreviations—EADs: early afterdepolarizations; DADs: delayed afterdepolarizations; AF: atrial fibrillation.

The most widely accepted pathomechanisms of AF are reentry and ectopic activity (Figure 1) [7, 13]. Both of these pathomechanisms are frequently the result of alterations in atrial tissue structure and function (or atrial remodeling), which can be induced by the disease or by AF itself. These pathomechanisms aid the progression towards increasingly persistent forms of AF [14, 15]. Abnormal local spontaneous discharges from overactive ectopic pacemakers cause ectopic activity. In normal atrial tissue, enhanced ectopy or reentry is rare, and these are often caused by the remodeling that takes place when cardiac diseases act on the tissue [15]. Delayed or early afterdepolarizations (repolarization failure) have also been suggested as the cause of ectopic activity [14]. Delayed afterdepolarizations (DADs) are caused by the simultaneous release of diastolic Ca^2+^ from the sarcoplasmic reticulum. This release generally occurs due to an overload of the sarcoplasmic reticulum [16] or dysfunction of the sarcoplasmic reticulum Ca^2+^ release channels [17–19]. Early afterdepolarizations (EADs) occur in response to excessive prolongation of action potential duration, which creates afterdepolarizations in the mechanisms that rely on Ca^2+^. EADs can also occur when short-duration action potential (or a parasympathetic effect) combines with prolonged transients of Ca^2+^. This is known as a sympathetic effect and occurs in response to sympathovagal discharge [20].

There are two methods of thinking about reentry. Allessie et al. [20] developed the leading circle concept, which theorizes that centripetal waves maintain this latter refractory through movement towards the center. The shortest circuit is the shortest distance an impulse travels in the refractory period, and it is in the shortest circuit through which a functional reentry is established. Following from the leading circle concept, a functional reentry depends on a balance between the speed of conduction and the refractory capabilities of the cells. The chances of simultaneous conduction occurring in a potential reentry area increase when there is a short refractory capacity or slow conduction speed. A spiral wave reentry, or a “rotor,” is another way in which reentry can take place. In this specific type, the reentry occurs in a particular region when a curved wavefront and wavetail come together at a point of a singularity. Additionally, this singularity's tissue must not be refractory [21]. In a spiral wave reentry, the reentry is achieved through rapid circulation around a central core by a rotor with a wavefront. To determine the size of the spiral wave, the excitability and refractoriness of the tissue of this reentry will be measured. If the tissue has a short refractory capacity and is highly excitable, rotor maintenance is achievable and can be stabilized, as these factors permit the spiral wave to rotate rapidly around a core.

The chances of reentry or ectopic firing are increased by remodeling (Figure 1). Remodeling encourages ectopic firing due to its alternating cardiomyocyte handling of Ca^2+^, which it does to encourage DADs or EADs to develop. Remodeling may be carried out electrically, but due to its rapid atrial rates, such as those seen when AF takes place, atrial refractoriness will be shortened by electrical remodeling through reduced action potential duration [21, 22]. AF has long-term effects on cellular structure, which remains remodeled (cardiomyocyte hypertrophy and glycogen accumulation). There is a possibility that assist rapid atrial activation promotes atrial fibrosis [23]. Atrial fibrosis may inhibit atrial conduction. If this occurs, an irreversible substrate for AF might be created.

Many pathologies are associated with AF, and a common feature amongst them is atrial fibrosis, which appears to play a central role in AF pathogenesis. Additionally, two features shared by many associated disorders—atrial strength and atrial enlargement—are key contributors to AF development. AF may be suppressed by an antiarrhythmic intervention, depending on whether or not the intervention has the capacity to suppress underlying mechanisms [23, 24].

## 3. miRNAs in AF

miRNAs have the potential to be appropriate disease biomarkers due to their tissue-specific and pathology-specific expressions. miRNAs are stable in plasma because of their ability to mingle with microparticles, such as exosomes, macrovesicles, and apoptotic bodies [25–27], and because they are frequently tied to proteins and high-density lipoproteins, which protect them from RNase activity. miRNAs also have high sensitivity and specificity and are detectable in both plasma and serum. Biomarkers can provide vital insight postdiagnosis [28]. When AF has been diagnosed, biomarkers can reveal atrial cardiomyopathies at the root of AF, which can have a widespread implication for prognosis and treatment. Such insights would certainly enhance patient care, making it more individually tailored. Numerous studies support the involvement of miRNAs in AF-related remodeling processes and have also suggested that miRNAs are likely to play vital roles in signaling during AF pathogenesis [29, 30].

### 3.1. Electrical Disturbances

Evidence suggests that increases in K+ current (*I*_K1_) and decreases in L-type Ca2+ currents (*I*_CaL_) are two of the most important ionic current changes at the base of AF-induced electrical remodeling. Multiple miRNAs have been identified in these types of remodeling, as well as in other components of electrical atrial remodeling [11]. miR-1 was first identified, in coronary artery disease, as having arrhythmogenic potential. It was also found to have a proarrhythmic effect in ischemic models because of the targeting of the gap-junction channel gene *GJA1* (encoding gap junction *α*1 protein) [31]. Furthermore, it has been shown that miR-1 is a regulator for Ca^2+^-handling proteins—for example, protein phosphatase 2A (PP2A), the Na^+^/Ca^2+^ exchanger 1, and calmodulin. However, additional studies are necessary to reveal whether AF is associated with miR-1-dependent regulation of Ca^2+^ handling. Another complication lies in the fact that the data implicates miR-26 as a regulator in AF changes in *I*_K1_ [32]. The calcium-/calmodulin-/calcineurin-regulated nuclear factor of activated T cells (NFAT) pathway has a negative control on the transcription of miR-26. Enriched NFAT nuclear translocation has been found in both dogs and in patients with AF [33]. This is likely to contribute to the reduction of miR-26. Still, these are not the only miRNAs involved in AF regulation. For instance, miR-208a is a crucial miRNA for cardiac hypertrophic responses. Spontaneous AF has been found to be frequent in miR-208a-knockout mice [34]. Furthermore, miR-328 has been found to be upregulated in AF patients [35], whilst miR-499 has been found to be upregulated through a miRNA expression study of atrial tissue in AF patients. This study compared AF patients with control participants [36].

### 3.2. Structural Remodeling

The trademark of structural remodeling in AF is atrial fibrosis. It is believed to have a crucial pathophysiological role in the condition, and miRNAs are considered to be potential regulators of the fibrotic remodeling that occurs in AF [11]. Of these miRNAs, miR-21 has a high expression of fibroblasts, and it has been intensively investigated through rodent models of cardiac hypertrophy. It is thought to target and repress the translation of the protein sprout homologue 1 (SPRY1) by encoding the protein. SPRY1 is a negative regulator of the extracellular signal-regulated kinase (ERK) pathway. Similarly, miR-26 may play an important role in AF-related electrical remodeling, but it is also thought to contribute to atrial fibrotic remodeling. It may participate in this remodeling by regulating the expression of transient receptor potential channel 3 (TRPC3) [32]. Furthermore, miR-29 is known to target multiple extracellular matrix genes, including collagen, fibrillin, and elastin [37]. miR-29b has been shown to be downregulated in the atria of dogs with heart failure. An inverse correlation has been found in its expression between its extracellular matrix protein levels and AF development [38]. In ventricular fibrosis during cardiac hypertrophy, miR-30 and miR-133 have been shown to be downregulated through the derepression of a vital profibrotic protein. Transforming growth factor- (TGF-) *β*1 and TGF-*β* receptor type-2 (TGFR-2) have also been discovered as profibrotic factors shown to be upregulated in nicotine-treated dogs. Reduced expressions of miR-133 and miR-590 (miRNAs that target TGF-*β*1 and TGFR-2) are also found in nicotine-treated dogs [39].

## 4. miRNAs as Potential Therapeutic Targets in AF

Recently, tissue-specific miRNAs have been studied in both humans and animals. These studies have implicated miRNAs as contributors to structural and electrical remodeling in AF [13, 40]. Changes in the expressions of miR-21, miR-26, miR-29b, miR-30c, miR-133, and miR-590 have been identified as having a relationship with AF structural remodeling. They are thought to regulate the signaling cascades related to atrial fibrosis. The changes in these miRNAs have also been found to be in relationship with electrical remodeling. miR-1 and miR-26, when downregulated, may help increase the basal inward rectifier current *I*_K1_. On the other hand, when miR-21 and miR-328 are upregulated, they appear involved in reducing L-type Ca2^+^ currents (*I*_CaL_) in the myocytes of patients with AF [35, 41]. Several relevant studies suggest that miRNAs are dysregulated in various AF forms, demonstrated by research in patients and animals.

### 4.1. Treatment of AF: The Role Played by Specific miRNAs, as Shown by Animal Studies

Tissue-specific miRNAs have been studied in animals, and various researchers have indicated that miRNAs play a crucial role in AF processes. *In vivo* manipulation of miRNAs in AF has been achieved, and the results suggest that particular miRNA therapeutics could be developed for atrial cardiomyopathies. Lu et al. [35] have discovered that antogomir-328 may successfully reverse AF susceptibility in ATP dogs through the *in vivo* adenoviral-mediated forced expression of miR-328. The capacity of miR-1 to reduce AF susceptibility has also been demonstrated by Jia et al. [42]. In this study, LNA-based antimir-1 was administered and found to prolong the atrial effective refractory period (AERP) and reduce AF susceptibility and duration in rabbits [43]. Furthermore, *in vivo* experiments in dogs and mice showed decreased Cav1.2, Cav*β*1, and *I*_CaL_, as well as shortening of the action potential duration and enhanced AF susceptibility [43]. The cluster of miR-106b-25 has been shown to be downregulated through upregulating ryanodine receptor 2 (RYR2) protein expression in patients with continual AF. Additionally, miR-106b-25 knockout mice have displayed a steady rise in Ca^2+^ release with RYR2, a known contributor to AF vulnerability [8]. Evidence shows that miR-29b expression is reduced in the atrial tissues of AF patients. These findings are supported by the observation of a miR-29b downregulation in the atrial tissues of canines undergoing ventricular tachypacing to induce congestive heart failure (CHF) and following AF [44].

### 4.2. Human Studies and What They Show about the Function of Particular miRNAs in AF Treatment

Various tissue-specific miRNA studies on human patients suggest that miRNAs play an important role in AF processes. When looking at the left atria (LA) of AF patients, a higher expression of miRNA-21 was seen compared to the LA of patients in sinus rhythm. A positive correlation was found between increased miRNA-21 expression and atrial collagen content. This relates to the reduced protein expression of SPRY1 and the increased expression of connective tissue growth factor (CTGF), lysyl oxidase, and Rac1-GTPase [45]. A recent study has shown that miR-21 and miR-150 have a relationship with AF, comparing 112 AF patients with 99 AF-free people [46]. In this study, the plasma levels of 86 miRNAs were measured. AF atrial remodeling pathogenesis was carried out for each miRNA by quantitative reverse transcription polymerase chain reaction (qRT-PCR). The levels of plasma in miR-21 and miR-150 were found to be noticeably lower amongst AF patients [46]. Another study was carried on both local and systemic plasma levels and found that miRNAs positively correlate with AF. Studies with atrial substrate properties suggest that miR-328 plays a vital role in atrial remodeling processes in AF patients [47]. miR-328 is locally produced in the LA. This may affect atrial remodeling in AF patients, as miR-328 plasma levels were higher in AF patients than in control patients. These levels were also measured as higher in the LA appendage than in the pulmonary vein (PV) or the periphery [47]. A study by Dawson et al. (2013) revealed that, in AF or congestive heart failure (CHF), patients' plasma exhibited a noticeable reduction of miR-29b and miR-21 expressions. This study further demonstrated that miR-29b was yet further decreased in AF and CHF patients [48]. CHF can cause fibrotic atrial remodeling and contribute to AF maintenance. Therefore, both of these miRNAs could be crucial biomarkers for atrial remodeling [5].

## 5. miRNA-Mediated Regulation of Inflammatory Cytokines in AF

Several studies have indicated that inflammatory mediators play a mechanistic role in AF pathophysiology. Inflammatory mediators such as C-reactive protein (CRP), interleukins, TNF-*α*, TGF-*β*, and MCP-1 were reported as having higher blood serum levels in AF patients than in control subjects. When miR-21 is upregulated, it promotes both AF and susceptibility to AF [8]. This is due to STAT3 phosphorylation or inhibition of the TGF-*β* pathway and downregulation of Smad7 [49]. Yet it is noteworthy that, *in vivo*, the control of miR-21 using anti-miR-21 has been shown to reduce AF and fibrosis in animals [50]. The CRP is also crucial to systemic inflammation. Additionally, a correlation of some strength is observed between miR-150 and CRP levels. When miR-150 is downregulated, it can aid AF growth through targeting genes that have a role in the inflammation [51]. Thus, it is a predictive biomarker for AF. Different cytokines—for example, TNF-*α*, TGF-*β*, IL-6, and IL-18 from macrophages and monocytes—are secreted in response to inflammatory stimuli. These produce increased plasma CRP production in AF patients but do not produce an increase in healthy subjects.

## 6. Prospective Future for miRNAs as Therapeutics

Research has demonstrated that there is a correlation between AF and quantifiable alterations in miRNA expression levels. Nonetheless, it cannot be ignored that differential miRNA expression levels, which have been measured through blood and tissue samples in the left and right atria, depend on the cardiac disease's severity or type. Furthermore, the phase and type of AF will impact the differential expression of miRNAs. These variables, alongside the methodologies used, should be considered when evaluating research. This must be done before miRNAs can be brought into clinical application [8, 43].

Despite the strength of the data supporting the effect of miRNAs in AF, the limitations of these studies cannot be overlooked. There are various inconsistencies, which are likely due to small sample sizes and variables (e.g., sex, age, drug therapies, and concomitant conditions). All these have influenced the studies to date. To clearly identify which miRNAs are dysregulated in clinical AF, further research is required. Further research is also required to determine what level of miRNA changes depends on the base pathology and the stage of the disease. Microarray techniques have been crucial to all studies profiling miRNA. These techniques are semiquantitative and are known for producing false-positive and false-negative results. Inarguably, future research using more quantitative methods, including high-throughput qPCR, is necessary. Research might also benefit from a fresh approach, such as deep sequencing, which would help researchers develop miRNA expression profiles in AF with precision and detail.

There are multiple concerns as to the safety of miRNA therapeutics, which would need to be addressed before miRNA-based therapy could be utilized in clinical practice. One of the primary concerns is miRNAs' ability to target multiple pathways. miRNAs might interfere with physiological pathways as a high number of miRNA mimics may be delivered to an organ that is not the target organ or a pathway not within the target tissue. Further research must be carried out to confirm the safety of miRNAs, as well as their therapeutic potential. Future research should, therefore, focus on the *in vivo* effects of cardiovascular miRNA therapeutics.
